# Pastoralism and Resulting Challenges for National Parks in Afar, Ethiopia

**DOI:** 10.1007/s10393-024-01687-6

**Published:** 2024-05-31

**Authors:** Samson Abebe, Hamere Melaku, Ashenafi GebreGiorgis Kidanu, Rea Tschopp

**Affiliations:** 1 Armauer Hansen Research Institute, Jimma Road, PO Box 1005, Addis Ababa, Ethiopia; 2 Ethiopian Wildlife Conservation Authority, Ras Abebe Aregay Street, PO Box 386, Addis Ababa, Ethiopia; 3 Department of Epidemiology and Public Health, Swiss Tropical and Public Health Institute, Kreuzstrasse 2, 4123 Allschwil, Switzerland; 4 University of Basel, Basel, Switzerland

**Keywords:** Ethiopia, Infectious diseases, Pastoralists, Wildlife–domestic animal interface, Wildlife threats

## Abstract

Pastoralists and national parks are key stakeholders in the management and conservation of natural and protected habitats. In Ethiopia, Afar pastoralists migrate seasonally with their livestock in search for grazing and water areas. Livestock are also a source of infectious diseases that can spread into wildlife populations when pastoralists encroach into unfenced national parks. The interactions between pastoralists and national parks, as well as the subsequent impacts, remain insufficiently understood in Afar. Two structured questionnaire surveys were conducted in 2021, including 300 pastoralist households in seven woredas of Afar, and 58 staff from three national parks (Awash, Alidegi and Yangudi Rassa). They captured pastoralist movements and livestock diseases as well as the perception of national park staff regarding challenges resulting from pastoral encroachment into parks. Among the pastoralists, 74.7% migrated with their livestock for a mean 3.5 months per year, during which time, 90% of respondents reported contact with other livestock herds, and over 80% with wildlife. A third (34.2%) reported disease outbreaks in their village prior to migration. Pastoralists traveled long distances, crossing woreda, regional or national boundaries. All 58-park respondents reported pastoralists with livestock inside their park and their close contact with wildlife. Additionally, 69% reported the presence of domestic dogs. Wildlife displacement, habitat loss and dog attacks on wildlife were perceived as the main threat caused by the presence of pastoralists, whereas diseases were only mentioned by 15.5%. Overall, park staff showed poor disease knowledge. They reported poor disease surveillance and no disease response. Within pastoral contexts, improved collaboration between wildlife and livestock authorities regarding land use, disease awareness and surveillance is needed to balance the needs of both wildlife and pastoralist’s livestock development and mitigate threats to wildlife habitats.

## Introduction

The Horn of Africa's economic sector greatly benefits from pastoralism (Fre and Tesfagergis, 2013). However, rangeland-based lifestyles and rangeland ecosystems are in danger and deteriorate over time. There are multiple causes for this, including the demand from an increasingly significant cash-based economy, a fast growing human population, dramatic weather fluctuations, animal diseases, overgrazing and anthropogenic land-use changes (Roderick et al., 1998; Admasu et al., 2010; Eldridge et al., 2016; Hussein et al., 2021).

Rangeland degradation is frequently associated with biodiversity loss (Alkemade et al., 2012; Belay et al., 2014; Selemani, 2020). Over the past few decades, research investigating the interactions between wildlife and livestock has expanded considerably. Nevertheless, there continues to be substantial controversy regarding the nature of wildlife–livestock relationships. Scholars from diverse fields have reported that domestic livestock compete with wildlife for natural resources (Young et al., 2005; Averbeck et al., 2009; Low et al., 2009). Others have indicated that livestock and wildlife can coexist without competition. In certain instances, the presence of livestock may even facilitate wildlife in accessing grazing areas that would otherwise be inaccessible to them (Sitters et al., 2009; Du Toit 2011; Du Toit et al., 2017). Among pastoral communities, people and their livestock may encounter wild animals during their seasonal migrations. Besides competition for natural resources, domestic animals can also spread viral, bacterial or parasitic infectious to wildlife and vice versa (Siembieda et al., 2011; Hassell et al., 2017; Hassel et al., 2020; Kagendo et al., 2021).

Diseases spread by humans, wildlife and domestic animals are posing an increasing challenge to public and animal health systems (Miller et al., 2013; Wiethoelter et al., 2015). Zoonotic diseases account for three-quarters of all human emerging infectious diseases (EIDs), with the majority of them originating in wildlife reservoirs (Kruse et al., 2004). Cross-species transmission is one of the least investigated areas of disease ecology, despite its importance (Jones et al., 2008).

Eastern Africa boasts some of the world’s highest densities and widest ranges of ungulate and mammalian species (Blake et al., 2008). Wildlife is essential to the economies of many countries, such as Kenya for instance, where tourism and wildlife viewing activities account for a major portion of the GDP (Waithaka, 2004). Livestock, on the other hand, are vital for sustaining rural and pastoral livelihoods on ecologically changing and varied rangelands (Scoones and Graham, 1994). While pastoralism is a land-use system that has the potential to be wildlife-friendly, there is a rising geographical overlap with a big proportion of wildlife located outside protected areas (Western et al., 2009). State and municipal policies related to conservation and livestock development have been impacted by poorly conceived understandings of these competitions. For instance, it can result in conflicting land-use policies that prioritize livestock development without considering ecological factors, leading to overgrazing, land degradation and habitat loss for wildlife. Conversely, if wildlife conservation is prioritized without considering the need of farmers, it can lead to conflicts and challenges for livestock husbandry. In the semiarid to arid lowlands of Ethiopia, pastoralism is a way of life adapting to the harsh environments, securing livelihood and making rational use of vulnerable drylands (Mohamed 2019). Pastoralists engage in seasonal migrations with their livestock in search of fodder and water, driven by dry seasons but also climatic shocks such as severe droughts, that have been more frequently observed in recent years.

The conservation and management of Ethiopia's national parks face numerous challenges: communities have limited awareness of the potential benefits of conservation, due to lack of awareness campaigns and benefit-sharing schemes, thus also missing a sense of ownership (Mekonen et al., 2017; Megaze et al., 2017). Additionally, Ethiopia faces a rapid population growth, which increases the demand for more farmlands, resulting in encroachment into wildlife habitats for natural resources (Temesgen and Warkineh 2018). This has put national parks under pressure. Communities have been settling in protected areas, illegally using the land for farming. On the other hand, pastoralists from different areas migrate seasonally through protected habitats in search for grazing land. Movement of pastoralists can potentially affect disease epidemiology in the national parks and affect wild animals. This has been demonstrated in the Bale National Park (Ethiopia), where rabies is endemic and has never been eradicated due to the migration of pastoralists with unvaccinated dogs (Osofsky and Cleaveland, 2005). Safeguarding both, people’s daily livelihood and wildlife conservation often remains challenging (Atuman et al., 2021). In Ethiopia, information on the pastoral-livestock-wildlife interface is rare. The main objectives of this study were therefore, to describe and understand, in light of infectious diseases prevalent in the Afar region of Eastern Ethiopia the mobility of pastoralists through the region, the implications this has for the interactions between pastoralists-domestic animals and wildlife and the perceived risks this poses to wildlife in national parks in Afar, through a pastoral and a national park lens.

## Materials and Method

### Study Area and Study Population

**Figure 1:**
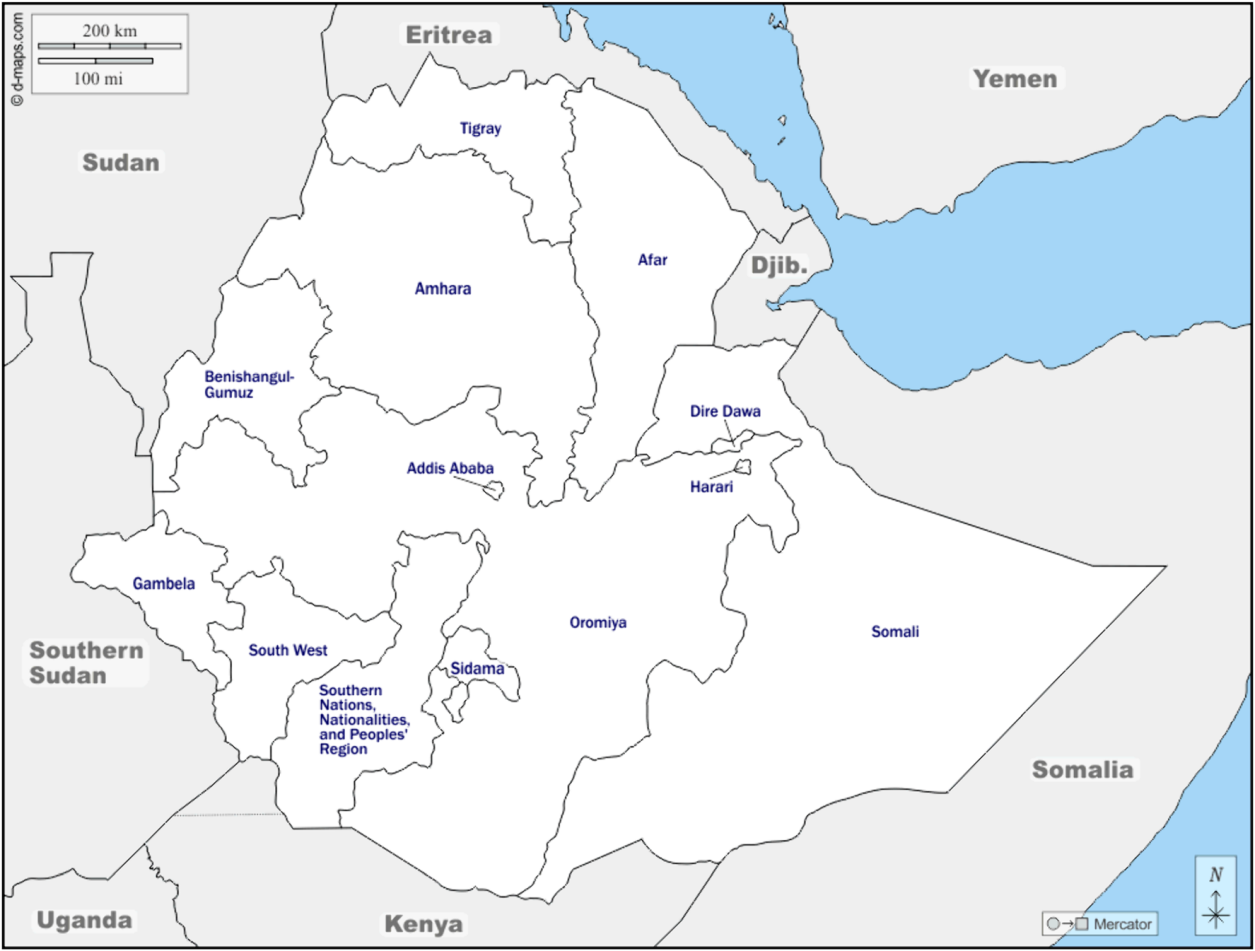
Map of Ethiopia showing the Afar region.

This study was part of a larger project assessing Brucellosis prevalence and its associated risk factors in Afar (Tschopp et al., 2021a). In addition to the referred seven *woredas* (district) chosen as study sites in the main project, we added three national parks in Afar (Fig. 1). Contiguous with one another, Awash National Park (598 km^2^) located 214.7 km and the Alidegi Wildlife Reserve (1832 km^2^) located 225 km East of Addis Ababa. Yangundi Rassa National Park (4730 km^2^), with its headquarter in Gewane, is located on the North-Eastern part of the country 365 km from Addis Ababa on the Djibouti road.

The Afar region is predominantly arid- to semiarid and faces severe recurrent droughts. It includes dry habitats characterized by savannah grassland, areas with sparse vegetation and riverine forests. It has a diversity of plant and animal species well adapted to these harsh conditions. Over 90% of the Afar community engage in pastoralist livelihood system, raising a mixture of livestock species (cattle, camel, goat and sheep) and often migrate seasonally. We refer to “migration” as the seasonal movement of people and their livestock between different grazing areas in search of water and pasture for their herds during dry seasons/droughts. This traditional system is ensuring the survival and well-being of their livestock. It also enables them to reduce overgrazing and land degradation in their home areas by allowing the land to recover during the absence of herds.

### Study Design

We conducted in that large project, a cross-sectional study to assess the knowledge, attitude and practice of brucellosis and anthrax among Afar pastoralists.

We refer to Tschopp et al (2021a, b) for the detailed study design. In summary, we conducted a multistage cluster sampling proportional to size, considering kebeles as cluster units. Households within kebeles were randomly selected from an official list of households owning livestock, which was provided by the kebele chairmen. In addition, we carried out a cross-sectional study from August to September 2021 in the three above described national parks. The rational for including these two different groups of participants was to obtain a more comprehensive and holistic understanding of issues and challenges faced by national parks and identify areas of conflicts between conservation goals and traditional practice of pastoralism. Both pastoralist and national park staff are key stakeholders in the management and conservation of national parks.

### Data Collection, Management and Analysis

Two structured questionnaires were prepared in English, translated into local language (afar language) and pre-tested. The first questionnaire was administered to pastoralists included in the overall study and captured overall demography, husbandry, disease knowledge, attitude and practice toward zoonotic diseases such as brucellosis and Anthrax (Tschopp et al., 2021b). We used a selected subset of this structured questionnaire for this present study, with questions pertaining specifically to livestock movement (e.g., trade, migration), contact with wildlife, perceived diseases and interactions with national parks. We administered the second questionnaire to national park staff in the selected three national parks. Questions captured information on geo-temporal animal–human interactions in national parks, involved animal species, description of the livestock–human–wildlife contact interface, perceived risks, disease knowledge, disease surveillance and response systems in place and the existence of a One-Health strategy in the area.

Each questionnaire received a unique numerical identification number for coding. All participants provided informed consents before enrollment in the study. Data was entered into Microsoft Access tables, with subsets of data copied into Microsoft Excel tables for further analysis. The data was analyzed using descriptive statistics using STATA-16 (StataCorp, Texas, USA).

This study received ethical clearance in Switzerland from the “Ethikkommission Nordwest-und Zentralschweiz” (EKNZ) (R-2017-000666) and institutional clearance at the Armauer Hansen Research Institute (AHRI), Ethiopia (P041-17).

## Results

A total of 300 pastoral households, and 58-park staff (management staff, experts, scouts) participated in this study.

### Pastoralist’s Survey

#### Livestock Movement

Livestock movements were reported during trading, with 72.3% (*N* = 217) and 13.3% (*N* = 40) pastoralists having sold or purchased livestock, respectively, in the last 12 months. Most trades were done with other districts.

**Figure 2:**
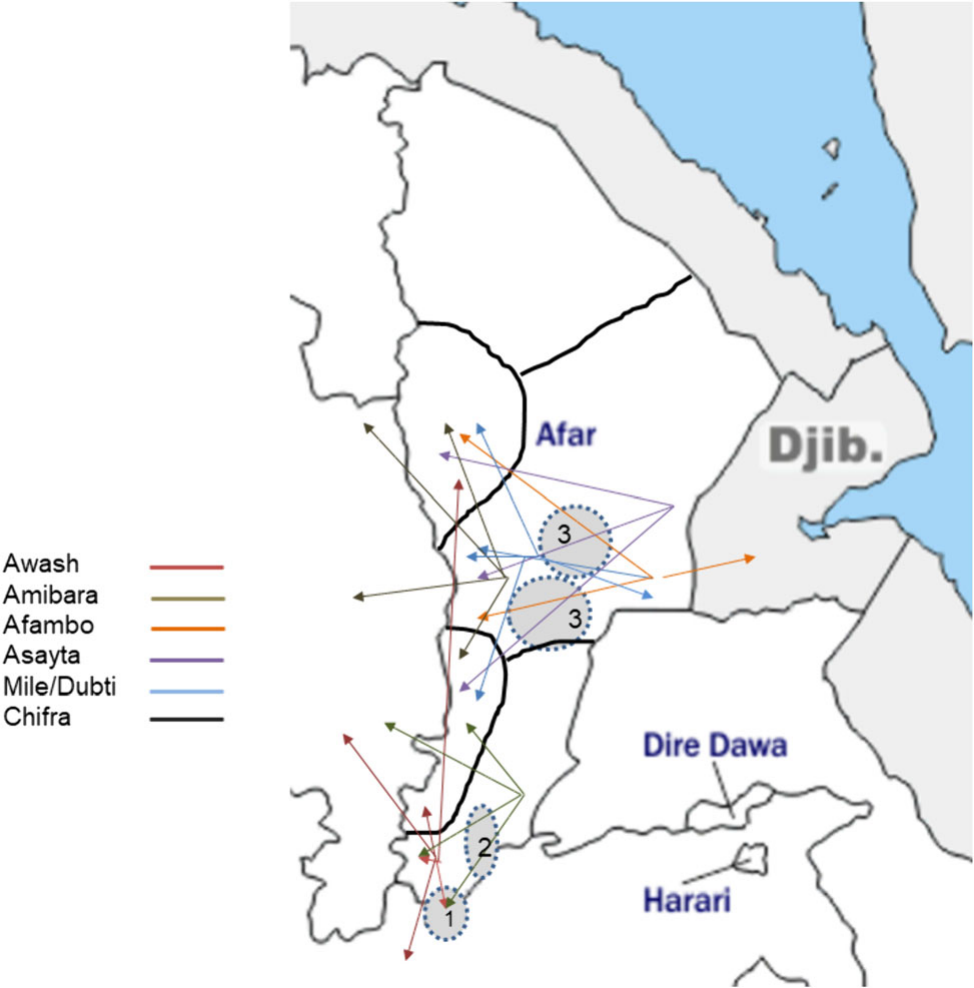
Sketch map showing the migration routes of surveyed pastoralists from seven woredas (routes are color coded by woreda; gray dotted circles represent national parks location with 1 = Awash, 2 = Alidegi, 3 = Yangudi Rassa).

The majority of respondents (*N* = 224; 74.7%) migrated with their livestock. Over half of them (*N* = 185; 61.7%) moved yearly, while 13% (*N* = 39) migrated occasionally depending on the year. Migrating pastoralists migrated with all their livestock species. Among the 224 migrating households, 223 (99.5%) would travel with cattle, 219 (97.8%) with camel, 215 (96.0%) with goats and 207 (92.4%) with sheep. Migration duration ranged between one and six months with a mean 3.5 months (SD = 0.89). Participants described the places they migrate to with their livestock (Fig. 2). They moved within their respective woreda but also beyond the woreda, regional or even national boundaries into neighboring Djibouti, covering large distances and by so doing, transiting through national parks.

The majority of pastoralists (*N* = 213; 95.1%) stated that their animals were mingling with other livestock herds during their migration.

#### Livestock Contacts with Wildlife and Diseases

The majority of the pastoralists (*N* = 256; 85.3%) stated that their livestock have contact with wildlife. Among them, 189 (63%) had occasional contacts, while 67 (22.3%) had contacts on a daily or weekly basis. When asked in an open question to list the most common wildlife species they encounter during their migration, most responded jackals (*N* = 255), followed by antelopes/gazelles (kudus, gerenuk, oryx, waterbucks; *N* = 244) (Table 1).

**Table 1:** Most common wildlife species observed by pastoralists during their migrations.

Category	Species	Number of households (%)
Carnivores		
	Jackal* (Lupulella mesomelas)*	255
	Hyena (*crocuta crocuta*)	104
	Spotted felids (serval cat, leopard)	3
	Lion (*Panthera leo*)	1
Antelopes/gazelles		
	Kudu, gerenuk, oryx	168
	Waterbuck (*Kobus ellipsiprymnus*)	76
Ostrich (*Struthio spp*)		92
Warthog (*Phacochoerus spp*)		60
Zebra (*Equus grevyi*)		59
Crocodile (*Crocodylus niloticus*)		4
Snakes		3
Hare		3

Overall, 75 migrating pastoralists out of 219 respondents (34.2%) observed disease outbreaks affecting livestock in their villages in the last 12 months. Among these outbreaks, the most commonly reported were pasteurellosis (*N* = 39), pox virus (*N* = 20), peste des petits ruminants (PPR) (*N* = 15) and Anthrax (*N* = 4). In addition, during that same period, we conducted a sero-surveillance of Brucellosis in the study area, which showed a prevalence in livestock (all species combined) ranging from 3.5 to 15.7% depending on the woreda (mean prevalence: 9%) (Tschopp et al., 2021b).

### National Park Survey

#### Pastoralists Occurrence

All park respondents (100%) stated that pastoralists come into their park with their livestock. Over half of them (56.2%) reported finding them in parks throughout the year, whereas the others responded they were observed season specifically (e.g., during dry season). The majority of pastoralists within the parks were believed to come from neighboring *kebeles* (*village*) (94.7%), and only few from other *woredas* within Afar and even other regions. The majority of respondents stated that pastoralists bring all their livestock (small and large livestock) into parks (*N* = 54, 93.1%), whereas another three respondents (5.2%) stated that they bring only large livestock (cattle and camels). Most park staff (82.8%) reported close contact between livestock and wildlife. This interface was mainly observed on grazing areas (*N* = 48; 82.7%), followed by the vicinity to water points (*N* = 26; 44.8%).

#### Problems Related to Pastoralists and Their Livestock in Parks

Overall, the majority of park staff responded that wildlife disturbance and displacement was the major problem resulting from pastoralists entering the park with their livestock (> 70%). However, in Awash National Park, staff reported clashes between pastoralists and park staff to be the main challenge (88.9%). Table 2 lists the main reported issues observed when pastoralists enter national parks and the perceived main threats to wildlife. Overall, the main threat to wildlife (51.7%) was habitat loss. This included habitat degradation, overgrazing, shrinking of grazing land and competition over grazing areas. Only 15.5% of the respondents mentioned diseases transmitted from livestock as an issue. A quarter (25.9%) of the interviewees had no opinions on possible existing threats to wildlife.

**Table 2:** Perceived problems encountered with pastoralists moving into parks and perceived main threats caused to wildlife.

		Overall (58)	Awash (27)	Alidegi (19)	Gewane (11)	HQ (1)
Problems encountered with pastoralists moving into Parks	Disturbed wildlife/change of behavior	42 (72.4)	21 (77.8)	12 (63.1)	7 (63.6)	1 (100)
	Displacement of wildlife	41 (70.7)	20 (74.0)	12 (63.1)	8 (72.7)	1 (100)
	Clashes between pastoralists and park staff	34 (58.6)	24 (88.9)	4 (21.0)	6 (54.5)	1 (100)
	Wildlife attack livestock	22 (37.9)	12 (44.4)	9 (47.4)	3 (27.3)	
	Livestock transmit diseases to wildlife	15 (25.9)	8 (29.6)	9 (47.4)	0	1 (100)
Main threats to wildlife	Habitat loss/degradation/loss of grazing land (including food competition, overgrazing, sharing grazing land)	30 (51.7)	12 (44.4)	9 (47.4)	8 (72.7)	1 (100)
	Stressed/disturbed wildlife, displaced wildlife (by people/livestock but also during ethnic clashes)	16 (27.6)	5 (18.5)	6 (31.6)	4 (36.4)	1 (100)
	Diseases transmitted from livestock (including consumption of dead livestock carcasses; fodder and water contamination)	9 (15.5)	3 (11.1)	3 (15.8)	2 (18.2)	1 (100)
	Human-wildlife conflict (including hunting/poaching, revenge killing for killed livestock)	7 (12.0)	7 (25.9)	0	0	0
	Conflicts between pastoralists & park staff	5 (8.6)	1 (3.7)	1 (5.3)	2 (18.2)	1 (100)
	Dog attacks	2 (3.4)	2 (7.4)	0	0	0
	Population decline	2 (3.4)	1 (3.7)	0	1 (9.1)	0
	None	2 (3.4)	2 (7.4)	0	0	0
	Pastoralist (in general, unspecified)	1 (1.7)	0	0	1 (9.1)	0
	Don't now	15 (25.9)	7 (25.9)	8 (42.1)	0	0

#### Disease Occurrence, Knowledge-Attitude and Practice in Parks

Over half (55.2%) of the respondents stated that there were no diseases in the parks. Of the listed diseases (Table 3), Anthrax was the most frequently mentioned (13.8%). Another 13.8% responded that they did not know about diseases. The majority of interviewees (86.2%) did not know about the source of these infectious diseases (Table 4), whereas 10.3% stated that diseases can be transmitted from livestock and 3.4% from dogs.

**Table 3:** Perceived diseases prevalent in national parks and their sources for infection.

Category		Overall (58)	Awash (27)	Alidegi (19)	Gewane (11)	HQ (1)
Diseases found in the parks	None	32 (55.2)	19 (70.4)	9 (47.4)	4 (36.4)	
	Anthrax	8 (13.8)	2 (7.4)	2 (10.5)	3 (27.3)	1 (100)
	Foot and mouth disease (FMD)	2 (3.4)		1 (5.3)	1 (9.1)	
	Bovine tuberculosis (BTB)	1 (1.7)	1 (3.7)			
	Contagious caprine pleuropneumonia (CCPP)	1 (1.7)		1 (5.3)		
	Rabies	1 (1.7)		1 (5.3)		
	Zoonosis	1 (1.7)			1 (9.1)	
	Parasites	1 (1.7)			1 (9.1)	
	No studies/no records	3(5.2)	1 (3.7)	1 (5.3)	1 (9.1)	
	Don’t know	8 (13.8)	4 (14.8)	4 (21.0)	0	0
Disease sources	within the National Parks (soil, water)	0	0	0	0	0
	Livestock	6 (10.3)	1 (3.7)	2 (11.8)	2 (18.2)	0
	Domestic dogs	2 (3.4)	1 (3.7)	0	1 (9.1)	0
	People	0	0	0	0	0
	Don’t know	50 (86.2)	25 (92.6)	17 (89.5)	8 (72.7)	1 (100)

**Table 4:** Information on disease outbreaks and disease knowledge (*N* = 58).

Diseases	Categories	Nb respondents (*N* = 58)	Specification
Rabies	Outbreaks in the last 12 months	56 (96.5)	No
		2 (3.5)	Yes
	Affected species	2 (3.4)	gazelles, rabbits, jackals, zebra
		56 (96.5)	Don’t know
	Outbreak frequency	1 (1.7)	2 month per year
		57 (98.3)	Don’t know
	Outbreak source	1 (1.7)	Wildlife
		57 (98.3)	Don’t know
	Mortality	1 (1.7)	5 animals/year
		57 (98.3)	Don’t know
Anthrax	Outbreaks in the last 12 months	53 (91.4)	No
		5 (8.6)	Yes
	Affected species	4 (6.9)	Gazelles, zebra, lesser kudu, gerenuk, oryx, warthog
		54 (93.1)	Don’t know
	Outbreak frequency	3 (5.8)	One time 10 years ago; last year; 4 years ago
		55 (94.2)	Don’t know
	Outbreak source	2 (3.4)	Livestock
		56 (96.5)	Don’t know
	Mortality	3 (5.8)	10 animals; 50 animals; > 7000 animals
		55 (94.2)	Don’t know
	Seasonality	2 (3.4)	No
		2 (3.4)	Yes, during wet season only
		4 (6.9)	Yes, during dry season only
		50 (86.2)	Don’t know
	Knowledge	5 (8.6)	Typical symptoms
		5 (8.6)	Told by livestock bureau
		1 (1.7)	Other
		38 (65.5)	Don’t know
Brucellosis	Knowledge	50 (86.2)	No
		8 (13.8)	Yes
	Source	4 (6.9)	Livestock
		54 (93.1)	Don’t know
	Transmission	2 (3.4)	Fecal contamination, contact at grazing/water areas
		56 (96.5)	Don’t know

Two-thirds (69%) of the respondents stated that domestic dogs were seen in national parks, either on a regular basis (10.3%) or sporadically (58.6%) (Table 5). Most dog encounters were observed in Awash National Park (77.8%) and the least in Yangundi Rassa National Park (45.5%). The origin of these dogs as whether they came from the park surroundings or with the migrating pastoralists differed by National Parks (Table 5). Overall, park staff stated that dog attacks on wildlife injuring and/or killing them was the main threat paused by dogs in parks (79.5%). Diseases were perceived as a lower threat to wildlife (20.4%). Only six respondents (10.3%) could name diseases transmitted by dogs, which included rabies (*N* = 6; 100%) and canine distemper virus (CDV) (*N* = 3; 33.3%).

**Table 5:** Dog population and their perceived threats to wildlife.

Dog population in Parks		Overall (58)	Awash (27)	Alidegi (19)	Gewane (11)	HQ (1)
Presence in Parks	No	17 (29.3)	5 (18.5)	6 (35.3)	6 (54.5)	0
	Yes, all the time	6 (10.3)	5 (18.5)	1 (5.9)	0	1 (100)
	Yes, occasionally	34 (58.6)	16 (59.3)	10 (58.8)	5 (45.5)	0
	Don’t know	3 (1.7)	1 (3.7)	2	0	0
Origin	Surroundings	24 (41.4)	9 (33.3)	9 (47.4)	4 (36.4)	1 (100)
	Accompany pastoralists	18 (31.0)	16 (59.2)	1 (5.3)	1 (9.1)	0
	Don’t know	16 (27.6)	2 (7.4)	9 (47.4)	6 (54.5)	0
Main threats for wildlife	None	4 (6.9)	0	0	4 (36.4)	0
	Attacks/kills	35 (60.3)	20 (74.1)	9 (47.4)	4 (36.4)	0
	Diseases	9 (15.5)	3 (11.1)	2 (10.5)	2 (18.2)	0
	Other	1 (1.7)	1 (3.7)	0	0	0

Table 4 shows the respondents answers regarding information on anthrax and rabies outbreaks in the parks as well as respondents’ knowledge about anthrax, rabies and brucellosis. Overall, response rates were very low due to lack of knowledge about these diseases (> 93%).

#### Disease Surveillance-Response and Sector Collaboration

We present overall compiled results due to the limited data available and similar results provided between the different sites. Overall, 53 respondents (91.4%) stated that there was no disease surveillance system in place. Among the four people who responded that there was one, following strategies were mentioned: patrolling and reporting dead animals; monthly monitoring. The majority of park staff (*N* = 52; 89.7%) stated that during disease outbreaks, samples were not sent to a laboratory for confirmation. The main reasons were a lack of qualified staff to collect samples (*N* = 34; 58.6%), lack of sampling consumables (*N* = 8; 13.8%) and lack of laboratories in the areas (*N* = 2; 5%). The other respondents did not know why. Twenty-seven (46.5%) of the park staff stated that carcasses were left out for scavengers, whereas 23 (39.6%) and 12 (20.7%) stated they would bury or burn them, respectively, and one respondent (1.7%) said carcasses would be covered up with branches. The majority (63.5%) did not know what happened to carcasses.

Similarly, 53 (91.4%) responded that there was no disease response in place. Two people (3.4%) said that the Ethiopian Wildlife Conservation Authority (EWCA) and the local district offices would be notified. One person added that responses could be challenging due to ongoing sporadic ethnic conflicts.

In an open-ended question, 55 participants shared their thoughts on how further disease outbreaks could be minimized or avoided (Table 6). Importance was put on management of domestic animals (54.5%), followed by improved surveillance and response strategies (49.1%), increased park human resources (27.3%) and improvement of the wildlife habitat (5.4%).

**Table 6:** Suggested improvements by respondents to avoid further disease outbreaks (*N* = 55).

Category		Number of respondents (%)	Total
Domestic animals	Vaccinate livestock	5	30
	Avoid dogs in the park	2	
	Medicinal plants tied around livestock’s neck	1	
	Community awareness (not to bring livestock into Parks)	23	
Surveillance and response	Increase monitoring/patrols for early disease detection	6	27
	Increase research in Park	4	
	Improve disease reporting	4	
	Availability of sampling consumables	3	
	Send samples to labs for confirmation	1	
	Better collaboration with kebeles, HQ and district livestock bureau	3	
	Swift removal of carcasses (burial/burning)	3	
	Kill sick animals	1	
	Medicate sick wildlife	2	
Human resources	Available Park veterinarians	14	15
	Strengthen Park management	1	
Habitat	Separate water areas for wildlife and livestock	2	3
	Ensure habitat quality	1	

The majority of park management staff and experts (60%) stated that meetings between park management and district livestock bureaus were very rare to inexistent (Table 7). The reasons for the lack of exchange were a lack of professional veterinarians in parks who could discuss with their livestock counterparts and diseases being a lesser concern in park management. This was also reflected by the topics discussed during these meetings with the major topic being issues related to land use (33.3%). However, 73.3% of the interviewees stated that a better collaboration between parks and livestock bureaus would be beneficial, for the following reasons: discuss preventive measures to avoid spillover of diseases from livestock to wildlife, minimize human-wildlife conflicts, share information and do more collaborative work and treat animals during outbreaks. The other respondents had no opinion on the matter.

**Table 7:** Collaboration with district livestock bureaus (*N* = 15), only park management staff and experts (not scouts).

Category		Overall (*N* = 15)
Meeting frequency with district livestock bureaus	Never	4 (26.7)
	Rarely (every couple of years)	5 (33.3)
	Once a year	2 (13.3)
	Several times a year	4 (26.7)
Discussion topics during meetings with sector bureaus	Land use	5 (33.3)
	People/wildlife conflict	4 (26.7)
	Disease outbreaks	4 (26.7)
	Disease surveillance/response	2 (13.3)

## Discussion

This study described pastoralist–wildlife interfaces in Ethiopia from the point of view of pastoralists and national park staff. Pastoralists have developed migratory or transhumant grazing strategies to mitigate their vulnerability to a limited resource base, particularly during droughts that often leads to high livestock mortality. The majority of pastoralists in our study (74.7%) were engaged in seasonal migration. They moved with their animals during a quarter of the year over sometimes, large distances, well beyond their resident district, crossing also regional or national borders into neighboring Djibouti for example. In addition, they moved also their animals for long distances to market places. Live animal markets are known to be typical hotspots for the spread of multiple infectious illnesses into new herds or new regions. While the hazards of bringing illness to a herd by purchasing animals are obvious, selling livestock offers a unique risk due to the bi-directional movement that occurs when not all animals are sold and then brought back into the initial herd (Motta et al., 2019). Movement of pastoralists over large distances, as seen in our study, is likely to also pose a risk for disease transmission into wildlife populations. Most pastoralists (85.3%) encountered wildlife during their migration, either inside national parks or outside protected habitats. They mentioned mainly antelopes and gazelle species, such as Oryx, gerenuks (*Litocranius walleri*) and kudus (*Tragelaphus* spp.), likely sharing grazing areas with their livestock. Waterbucks (*Kobus ellipsiprymnus*) were also frequently observed. This species is typically found near water bodies, also potentially shared by livestock. Grevyi zebra (*Equus grevyi*) were also listed among the frequent encounters. They are, together with the Beisa Oryx (*Oryx beisa*), two endangered species in Ethiopia, hence at risk of infectious diseases transmitted by livestock, particularly Anthrax (Muoria et al., 2007; Low et al., 2009). Carnivores, specifically jackals (*Lupulella mesomelas*), were also mentioned to come close to their livestock. In a previous study from Somali region, pastoralists reported that jackals were perceived to be linked to rabies outbreaks (Ibrahim et al., 2022).

National parks are not fenced in Ethiopia, thus allowing for in and out movements of people, domestic animals, and wildlife and consequently a close interface of actors. All of the interviewed park staff (100%) reported that pastoralists come into parks with their livestock in search for grazing area and/or water. They were thought to come mainly from neighboring *kebeles* (94.7%). However, pastoralist’s description of their migration routes points also to much longer distances, unbeknown to park staff. The majority of the park respondents (82.8%) reported close interaction between livestock and wildlife, mostly on grazing areas, but also around water points. This setting favors diseases transmission, as well as habitat degradation through overgrazing and displacement of wildlife. Habitat degradation was reported by the park staff to be the top threat to wildlife (51.7%), whereas risk for diseases was only stated by 15.5% of the respondents. When cattle population concentrations exceed the natural environment's acceptable carrying capacity, the ecosystem is degraded, making it unsuitable for the existing wild herbivores living in the area (GebreMichael et al., 1992; Kebede et al., 2014). Through extensive uptake of palatable plant species, livestock grazing can also result in the reduction of primary fodder, decline of woody species richness and facilitate the proliferation of invasive and unpalatable plant species (Johnson et al., 2010; Eldridge et al., 2016; Marino et al., 2017). This situation has been reported in and around the Bale Mountains National Park in Central Ethiopia, affecting the availability and quality of feed for mountain Nyalas (*Tragelaphus buxtoni*) (Johnson et al., 2010).

Regular ethnic conflicts in Afar around National Parks (between Kereyu and Afar or Afar and Issa ethnic groups) have been described by the study respondents also to be detrimental to wildlife, leading to wildlife population displacements. The effect of armed conflict on wildlife populations in Ethiopia has so far been poorly studied.

Two-thirds of the park staff (69%) reported also dogs within the park boundaries, either accompanying pastoralists or coming by themselves from surroundings villages. Although the possibility of disease transmission was mentioned, the biggest threat posed by dogs to wildlife was perceived to be rather dogs attacking, injuring and/or killing wildlife as reported by 79.5% of the respondents.

This study showed that park staff overall perceived diseases as a risk to wildlife to be very low. Diseases, however, pose a real threat to wildlife. In the Bale Mountain National Park and in Delanta (Wollo), domestic dogs were associated with regular rabies outbreaks in the endemic Ethiopian wolf (*Canis simiensis*) population, resulting in severe population reduction (Sillero-Zubiri et al., 1996; Marino et al., 2017). A study in Kenya showed that outbreaks of rinderpest in the mid-1990s on shared grazing land resulted in mortality as high as 60% in buffalo and 90% in kudus in some areas (Osofsky et al., 2005). Moreover, our previous work and the work of other researchers have highlighted the prevalence of several zoonotic diseases among pastoral communities in Afar, such as bovine tuberculosis (Berg et al., 2015; Tschopp et al., 2015), brucellosis (Zerfu et al., 2018; Tschopp et al., 2021a), Anthrax (Tschopp and Kidanu, 2024) and rabies (Tschopp et al., 2016). All of them posing a potential threat to wildlife species (Simpson et al., 2021; Aruho et al., 2021). A third of the interviewed pastoralists came from areas where animal disease outbreaks had occurred before migrating.

Poor disease knowledge has commonly been reported in previous studies among pastoral communities in Ethiopia and abroad (Kiffner et al., 2019; Özlü et al., 2020; Tschopp et al., 2021b). Our study highlighted the very poor disease knowledge also among park staff, regardless their working position. Only eight people (13.7%) stated that domestic animals could be a source for diseases. Few diseases were mentioned such as anthrax, foot and mouth disease, rabies and contagious caprine pleuropneumonia (CCPP) but 86.2% did not know the source and transmission of these diseases, and over 93% of the respondents did not know about the epidemiology of anthrax, rabies or brucellosis. This highlights the urgent need for better disease awareness among all park staff.

Disease surveillance systems as well as outbreak responses were lacking or inexistent in all three national parks. Few measures included patrolling and reporting dead animals. However, the majority of respondents did not know what was happening to carcasses. Biological samples were never collected, hence no laboratory confirmation performed. The main constraints for sample collection in parks were primarily a lack of qualified veterinarians in parks (58.6%), followed by shortage of sampling equipment and the inexistence of nearby laboratories. Although tackling the domestic animal issue (e.g., vaccination, community awareness) was seen as the top priority of most respondents (*N* = 30; 54.5%) in order to avoid future disease outbreaks, regular discussions between park management and district livestock bureaus were said to be rare to inexistent (60%). However, two-thirds of the interviewees recognized the importance of a strengthened collaboration between park staff and livestock bureaus. This study could unfortunately not assess the existing bottlenecks for such One-Health collaboration.

## Conclusion

This study highlighted the need for a better understanding of the disease epidemiology at the livestock-wildlife interface in Afar, for improved disease awareness and professional training among national park staff and for increased coordination between the livestock, human and wildlife sectors involving joint disease surveillance and control at interface sites following One-Health approaches. Disease control in pastoral areas needs to be tailored to- and account for pastoral mobility. Better knowledge of migration routes may help improving preventive measures. Pastoralists are intricately linked to the ecosystem in which they live and to the livestock they breed for daily livelihood, but they can also play a significant role in the conservation and sustainable use of rangeland. Integrated sustainable approaches are, therefore, needed to balance the needs of both wildlife conservation and livestock development.
